# Key Laboratory Markers for Early Detection of Severe Dengue

**DOI:** 10.3390/v17050661

**Published:** 2025-04-30

**Authors:** Kumar Sivasubramanian, Raj Bharath R, Leela Kakithakara Vajravelu, Madan Kumar D, Aritra Banerjee

**Affiliations:** 1 Department of Microbiology, SRM Medical College Hospital and Research Center, SRM Institute of Science and Technology, Kattankulathur, Chengalpattu 603203, Tamil Nadu, India; ks1063@srmist.edu.in (K.S.); leelav@srmist.edu.in (L.K.V.); md5398@srmist.edu.in (M.K.D.); ab0452@srmist.edu.in (A.B.); 2 Department of Transfusion Medicine & Blood Centre, SRM Medical College Hospital and Research Center, SRM Institute of Science and Technology, Kattankulathur, Chengalpattu 603203, Tamil Nadu, India

**Keywords:** severe dengue, platelet transfusion, platelet count, activated partial thromboplastin time, hospital stay

## Abstract

Dengue virus is the most prevalent arthropod-borne viral disease in humans. Severe dengue, defined by hemorrhagic fever and dengue shock syndrome, can develop quickly in people who have warning indications such as abdominal pain, mucosal bleeding, and a significant decrease in platelet count. Laboratory markers such as hematocrit, platelet count, liver enzymes, and coagulation tests are critical for early diagnosis and prognosis. This retrospective study was carried out from January 2023 to December 2024 at a super-specialty tertiary care hospital. There were 283 adult patients with dengue with warning signs, who were categorized into 102 with platelet transfusion and 181 with no platelet transfusion. Data on patient demographics, clinical history, laboratory values, and radiological findings were systematically obtained from hospital records at the time of admission. Laboratory parameters such as white blood cell (OR = 2.137), hemoglobin (OR = 2.15), aPTT (OR = 5.815), AST^2^/ALT (OR = 2.431), platelet count (OR = 26.261) and NS1 (OR = 4.279) were found to be significantly associated (*p* < 0.01) with platelet transfusion. Similarly, an increased prothrombin time (OR = 2.432) contributed to prolonged hospital stays and the presence of ascites (OR = 5.059), gallbladder wall thickening (OR = 4.212), and pleural effusion (OR = 2.917), contributing to the severity of the dengue infection. These significant laboratory markers help with identifying patients with dengue who may develop severe dengue, requiring platelet transfusion, thereby prioritizing patient care and enabling the implementation of targeted interventions.

## 1. Introduction

Dengue virus is the most common arthropod-borne viral illness in humans. It belongs to the Flaviviridae family and consists of four dengue serotypes: Denv1, Denv2, Denv3, and Denv4 [1,2]. According to the 2009 WHO (World Health Organization) guidelines, the warning signs of dengue are persistent vomiting, clinical fluid accumulation, mucosal bleeding, fatigue or restlessness, liver enlargement greater than 2 cm, abdominal pain, and an increase in hematocrit accompanied by a rapid decrease in platelet count, indicating a risk of severe dengue [3]. Severe hemorrhagic fever and dengue shock syndrome (DSS) can rapidly develop in patients with dengue, particularly when they present with one or more dengue warning signs. These conditions represent the severe manifestations of dengue, where the disease can escalate quickly to life-threatening stages, and patients may require platelet transfusion [4]. Laboratory indicators have an important role in the early detection and prognosis of dengue in patients exhibiting warning signs. These markers include hematological markers (e.g., hematocrit, platelet count), biochemical markers (e.g., liver enzymes), activated partial thromboplastin time (aPTT), prothrombin time (PT), and other specific indicators [5,6,7]. Certain methods such as abdominal ultrasonography are effective for detecting dengue-related vascular leakage and organ involvement by identifying ascites, splenomegaly, hepatomegaly, and gall bladder wall thickening [8,9]. While platelet transfusions are commonly used to manage severe thrombocytopenia and hemodynamic changes in patients with dengue, their necessity and clinical benefits remain uncertain, as transfusion decisions depend on various factors beyond platelet count alone, although bleeding symptoms in patients dengue are considerably variable and may not consistently correlate with the severity of the disease. Various clinical and laboratory indicators have been investigated to predict outcomes in severe dengue. The severity of the dengue in a patient can be determined by the warning signs as well as bleeding severity and transfusion requirements, which can happen in severe dengue, leading to prolonged hospital stay. The evaluation of these laboratory indicators provides critical insights into disease severity, complication risks, and the necessity of platelet transfusion, facilitating the evaluation of markers in patients with dengue exhibiting early indicators to predict severe dengue.

## 2. Materials and Methods

**Figure 1:**
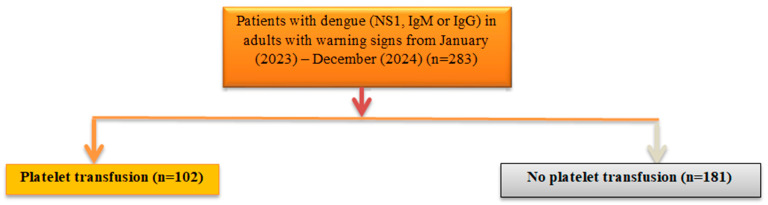
Flowchart for platelet transfusion in patients with dengue with warning signs.

This retrospective study was conducted from January 2023 to December 2024 at a super-specialty tertiary care hospital, involving a total population of 976 patients who tested positive for dengue. A total of 283 adult patients with dengue exhibiting warning signs were analyzed, with 102 receiving platelet transfusions and 181 not receiving platelet transfusions in Figure 1. The Institutional Ethics Committee (clearance number: 8684/IEC/2023) approved this study, verifying that ethical norms were followed.

This study’s data contained detailed patient information, clinical histories, and routine laboratory parameter test results at the time of admission; patients were followed-up if they had received a platelet transfusion. All information was systematically extracted from the hospital’s medical records to permit a thorough investigation of the clinical and laboratory characteristics of these patients. From, this study examined three dependent variables, platelet transfusion, bleeding, and length of hospital stay, to analyze their correlation with laboratory parameters and other clinical indicators in patients with dengue exhibiting warning signs.

### Statistical Analysis

Continuous variable are presented as mean and SD, and categorical data are expressed as frequency and percentage. Levene’s independent *t* test was used to test for differences in continuous variables, and the chi-square test was used to assess the differences between categorical variables. A *p* value of <0.01 was considered statistically significant. Then, the risk was examined using cross-tabulation and Fisher’s exact test to find the odds ratio. All analyses were performed using statistical software, SPSS, version 25.0.

## 3. Results

Descriptive data were analyzed for different variables including age, sex, comorbidities, clinical features, WBC, PCV, Hb, AST, ALT, AST^2^/ALT, aPTT, PT, platelet count, hospital stays for those who received platelet transfusion (*n* = 102) and those who did not receive a platelet transfusion (*n* = 181), bleeding (*n* = 43), no bleeding (*n* = 240), hospital stays of more than 6 days (*n* = 82) and hospital stays of less than 6 days (*n* = 201). The results are shown in Table 1.

**Figure 2:**
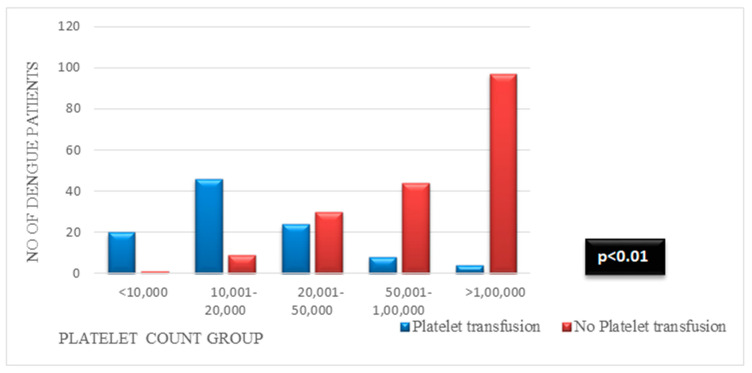
Different categories of platelet count for those who received platelet transfusion or not.

Figure 2 shows the distribution of the platelet counts (cells/mm) between patients with dengue receiving platelet transfusion versus those who did not receive platelet transfusion. The Y axis displays the number of patients in each category. There was a significant difference between the groups (*p* < 0.01).

The correlation of the independent variables, including the numerical mean of the laboratory values of the WBC, Hb, AST^2^/ALT, aPTT, PT, and platelet count) and categorical data (NS1, ascites, gall bladder wall thickening/edema, and pleural effusion), with the dependent variables (platelet transfusion, bleeding, and length of hospital stay) formed the categorical data in the cross-tabulation (2 × 2), which were used to calculate the odds ratio.

As shown in Table 2, platelet count (OR = 26.261) was a highly significant predictor, underscoring the crucial role that severe thrombocytopenia plays in severe dengue cases needing transfusion.

Between those with bleeding and no bleeding, we found statistically significant differences in the odds ratio for aPTT (*OR* = 3.074) and platelet count (*OR* = 8.352); the strong correlation between low platelet count and bleeding risk emphasizes the need for platelet monitoring (Table 3).

Following the of the length of hospital stay (>6 days and <6 days) found statistical significant difference with Odds ratio only in PT (*OR* = 2.432) (Table 4).

## 4. Discussion

The purpose of this study was to identify the predictors of the need for platelet transfusion and bleeding risk in patients with dengue with warning signs when admitted to the hospital, as these factors contribute to prolonged hospital stays. An analysis comparing patients who received platelet transfusions with those who did not receive platelet transfusions showed significant differences in several clinical and laboratory parameters. In patients who received a platelet transfusion, the following laboratory parameters, s ascites (41.2%), gall bladder wall thickening/edema (30.4%), pleural effusion (25.5%), packed cell volume (PCV) (42.5%), hemoglobin levels (14.4 g/dL), activated partial thromboplastin time (aPTT) (37.2 s), AST^2^/ALT levels (338.5 U/L), platelet count (26,473.5 cells/mm^3^), NS1 antigen positivity (91.17%), and IgM and IgG positivity rates (35.29% and 12.74%, respectively) were significant in comparison to those in patients with no platelet transfusion. These laboratory markers may serve as indirect indicators of disease severity, which, in turn, influence transfusion decisions. Notably, 25 patients (24.5%) received platelet transfusions despite having a platelet count exceeding the mean transfusion threshold, suggesting possible irrational transfusion practices. Prashantha et al. (2014) reported a higher rate (35%) of unnecessary platelet transfusions in stable patients with dengue, reinforcing concerns about the overuse of prophylactic platelet transfusions without clear clinical indications [10].

The length of hospital stay was notably longer in patients requiring platelet transfusion, at more than 6.5 days. Similarly, comparing patients with and without bleeding events, the platelet count was significantly lower in those who experienced bleeding. Furthermore, hospital stays exceeding six days were significantly associated with prolonged prothrombin time (PT) (15.5 s), *p* < 0.01. The findings from this study align with those of Lee T-H et al., who reported significantly longer hospital stays in patients requiring platelet transfusions (6 days vs. 5 days, *p* < 0.001) [11]. Similar studies have noted an extended hospital stay in patient who received transfusions, although without the other parameters being statistically significant [12]. Our analysis also demonstrated a significant association between platelet transfusion and prolonged hospitalization. The study findings regarding hospital stays of more than 6 days were statistically significant and correlated well with other study findings. In our study, we found only 10 patients who spent more than 10 days in the hospital. So, we are unable to perform further analysis.

Several factors may contribute to this prolonged hospitalization, including persistent thrombocytopenia, ongoing bleeding episodes, organ involvement such as liver dysfunction, and secondary infections. Additionally, transfusion-related complications and inappropriate platelet transfusion practices may delay recovery. A low white cell count (3.8 × 10^9^/L) was associated with inadequate platelet recovery, but the risk estimation was low (OR = 0.83) [11]. Dengue virus infection contributes to lymphocyte depletion, through both direct viral effects and inflammatory cytokines, leading to leukopenia and immunosuppression, which may worsen disease progression [13].

PCV was a highly significant predictor in our study, consistent with the findings of Nandwani et al., who identified an elevated PCV (≥45%) in conjunction with a low platelet count (<20,000 cells/mm^3^) as indicators of plasma leakage and dehydration (OR = 2.32). Hemoconcentration due to fluid loss is a hallmark of severe dengue, reinforcing the importance of PCV in predicting disease severity [14]. Our findings indicate that elevated hemoglobin levels (>14.3 g/dL) were associated with an increased severity of dengue (39.2%, OR = 2.151).

In line with Arshad et al., who reported NS1 antigen positivity in 85.5% of cases and IgG/IgM positivity in 34% [15], our study found a high prevalence of NS1 antigen positivity (91.17%) in patients requiring platelet transfusion. Additionally, the prevalence was similar in bleeding cases, suggesting NS1 antigen is a crucial marker for early dengue identification and severity prediction. Early NS1 testing is essential for timely therapeutic intervention. IgM positivity was highly significantly (0.003) correlated with platelet transfusion in our study, indicating immune activation. Srisuphanunt et al. found IgM positivity (33.8%) and IgG positivity (43.6%) in patients with dengue, with risk estimated for IgM at OR = 2.407. Our findings suggest that IgM plays a role in early immune response activation [16]. The World Health Organization (1999) guidelines emphasize the importance of NS1 antigen, platelet count, and coagulation parameters in stratifying dengue severity. Incorporating these markers into clinical judgment can enhance the early detection and management of severe cases [3].

Our findings corroborate prior research highlighting the predictive value of radiographic and laboratory indicators in people with severe dengue. We found a significant association between pleural effusion (OR = 2.917) and severe dengue, aligning with Shabbir et al. (2018), who identified pleural effusion (12.6%) as a marker of severe disease [17]. However, Lee et al. (2016) did not find pleural effusion to be a significant predictor [18]. Motla et al. (2011) emphasized the role of ultrasound findings, including regarding ascites (74.6%), pleural effusion (28.4%), and gall bladder wall thickening (GBWT) (72%), in predicting dengue severity [19]. According to Dayanand KR et al., gall bladder wall thickening (86.5%) and ascites (41.7%) are the most prevalent ultrasonography findings in seropositive patients [20]. Another study found that 89.9% of patients with DHF had gall bladder walls thicker than 3 mm [21]. Yuan K et al. found a high significance according to the SD between pleural effusion and ascites (OR = 15.84 and OR = 24.29) [22]. Our study found that 41.2% of patients had ascites (OR = 5.059), 30.4% had gall bladder wall thickening (OR = 4.21), and 25.5% had pleural effusion (OR = 2.917), which caused plasma leakage and platelet transfusion, but Ibrahim et al. (2022) found that only GBWT (OR = 21.7) was a more significant predictor of severe dengue than the dengue warning signs [9]. These findings were primarily observed in pediatric patients, whereas our study identified these markers in adults with dengue warning signs. Additionally, hepatomegaly was found to be weakly significant (0.024) in patients requiring platelet transfusion.

Our study also found a significant association between the AST^2^/ALT levels and platelet transfusion outcomes (OR = 2.431) at a threshold of 169.3. The AUC analysis by Md sani et al. suggested a threshold of 402.5 for predicting severe dengue. However, discrepancies exist in the association between AST^2^/ALT and severe dengue, particularly regarding prolonged hospitalization [23].

Malhi et al. identified significant predictors of prolonged hospital stays (>3 days), including hemorrhagic fever (16.2%), elevated alkaline phosphatase (ALP) (27.1%), prolonged PT (43.9%), prolonged aPTT (32.6%), and multi-organ dysfunction (22.3%) [24]. Although our study found prolonged PT (39.6%) to be a significant factor, the other parameters were not associated with extended hospitalization. Notably, no mortality predictors were identified in our cohort. Hospitalization duration is a critical factor in resource allocation and healthcare burden assessment. Our study found that PT (OR = 2.432) was a strong predictor of prolonged hospital stays (>6 days), suggesting that coagulation abnormalities may necessitate extended inpatient care.

Our findings indicate a strong association between platelet count and bleeding risk at a specific cutoff point (<26,179.07 cells/mm^3^, OR = 8.352) at the time of admission. Chi et al. (2023) found that an elevated aPTT (>50 s) (36.2%) was associated with a high bleeding risk (OR = 14.88). Liver enzymes (>200 U/L) (25.5%) were also linked to increased risk (OR = 7.39) [25]. Our findings, however, identified platelet count and aPTT as the primary predictors, while AST and ALT were not significantly associated with bleeding risk. The strong correlation between a low platelet count and bleeding underscores the need for continuous platelet monitoring in patients with dengue according to our findings. Similar to our results, Logia et al. (2023) and Thomas et al. (2024) identified thrombocytopenia and prolonged aPTT as significant bleeding risk factors. Dengue-related bleeding is multifactorial, driven by thrombocytopenia, coagulopathy, and endothelial dysfunction, which disrupt the balance of the procoagulant and anticoagulant systems, leading to prolonged aPTT [26,27].

## 5. Conclusions

This study highlights key diagnostic and laboratory markers essential for the early identification of high-risk patients with dengue. These significant predictors include platelet count and aPTT, which are crucial for assessing platelet transfusion requirements and bleeding risk at the time of admission. Additionally, NS1 antigen positivity, the presence of ascites, gall bladder wall thickening, and pleural effusion serve as reliable indicators of platelet transfusion needs, while an elevated PT is associated with prolonged hospitalization. These findings emphasize the importance of early monitoring and intervention using readily available laboratory parameters that are routinely collected at the time of admission to a hospital. By identifying high-risk patients early, healthcare providers can improve patient outcomes, optimize resource allocation, and ensure timely management of those exhibiting the warning signs of severe dengue.

## Figures and Tables

**Table 1:** Descriptive data analysis and clinical features at the time of admission.

Variable	Platelet Transfusion *(n* = 102)	No Platelet Transfusion (*n* = 181)	Bleeding (*n* = 43)	No Bleeding (*n* = 240)	Hospital Stay (>6 Days) (*n* = 82)	Hospital Stay (<6 Days) (*n* = 201)
**Sex (%)**						
**Male**	62 (60.8)	117 (64.6)	22 (51.2)	157 (65.4)	50 (61)	129 (64.2)
**Female**	40 (39.2)	64 (35.4)	21 (48.8)	83 (34.6)	32 (39)	72 (35.8)
***p* value**	0.518 ^a^		0.074 ^a^		0.612 ^a^	
**Age (mean ± SD)**	30.5 ±12.1	32.01 ± 14	31.7 ± 14	31.42 ± 13.3	32.52 ± 14.8	31.03 ± 12.8
**<20**	19 (18.6)	42 (23.2)	10 (23.2)	51 (21.3)	17 (20.7)	44 (21.9)
**21–30**	48 (47)	64 (35.3)	16 (37.2)	96 (40)	30 (36.6)	82 (40.8)
**31–40**	16 (15.6)	35 (19.3)	5 (11.6)	46 (19.2)	16 (19.5)	35 (17.4)
**41–50**	12 (11.7)	12 (6.6)	7 (16.3)	17 (7.1)	9 (11)	15 (7.4)
**51–60**	4 (3.9)	18 (10)	3 (7)	19 (7.9)	3 (3.7)	19 (9.5)
**>61**	3 (2.9)	10 (5.5)	2 (4.6)	11 (4.6)	7 (8.5)	6 (3)
***p* value**	0.094 ^a^		0.420 ^a^		0.173 ^a^	
**Comorbidities (%)**						
**Diabetes**	13 (12.7)	19 (10.5)	6 (13.9)	26 (10.8)	16 (19.5)	16 (8)
**Hypothyroidism,**	3 (2.9)	10 (5.5)	2 (4.6)	11 (4.6)	2 (2.4)	11 (5.5)
**systemic**						
**Hypertension**	3 (2.9)	10 (5.5)	1 (2.3)	12 (5)	6 (7.3)	7 (3.5)
**Anemia**	1 (1)	5 (2.8)	-	6 (2.5)	2 (2.4)	4 (2)
**Clinical features (%)**						
**Abdominal pain-**	13 (12.7)	108 (59.7)	-	121 (50.4)	28 (34.1)	93 (46.3)
**Fluid accumulation**	20 (19.6)	34 (18.8)	-	54 (22.5)	15 (18.3)	39 (19.4)
**Rapid decrease in platelets with rise in hematocrit**	16 (15.7)	7 (3.9)	-	23 (9.6)	9 (11)	15 (7.5)
**Persistent vomiting**	11 (10.7)	31 (17.1)	-	42 (17.5)	12 (14.6)	30 (14.9)
**Radiology findings**						
**Ascites (%)**	42 (41.2)	22 (12.1)	16 (37.2)	48 (20)	19 (23.2)	45 (22.4)
***p* value**	**0.000 ^a^**		0.013 ^a^		0.886 ^a^	
**Gall bladder wall thickening (%)**	31 (30.4)	17 (9.4)	12 (27.9)	36 (15)	10 (12.2)	38 (18.9)
***p* value**	**0.000 ^a^**		0.038 ^a^		0.172 ^a^	
**Splenomegaly (%)**	13 (12.7)	16 (8.8)	1 (2.3)	28 (11.7)	10 (12.2)	19 (9.4)
***p* value**	0.298 ^a^		0.063 ^a^		0.490 ^a^	
**Pleural effusion (%)**	26 (25.5)	19(10.5)	12 (27.9)	33 (13.7)	15 (18.3)	30 (14.9)
***p* value**	**0.001 ^a^**		0.019 ^a^		0.482 ^a^	
**Hepatomegaly (%)**	16 (15.7)	13 (7.2)	7 (16.3)	22 (9.2)	9 (11)	20 (9.9)
***p* value**	0.024 ^a^		0.157 ^a^		0.796 ^a^	
**Blood group (%)**						
**A**	12 (17.6)	34 (18.8)	7 (16.3)	39 (16.2)	15 (18.3)	31 (15.4)
**B**	40 (33.3)	59 (32)	14 (32.5)	85 (35.4)	29 (35.4)	70 (34.8)
**AB**	6 (5.9)	13 (7.7)	1 (2.3)	18 (7.5)	1 (1.2)	18 (8.9)
**O**	44 (43.1)	75 (41.4)	21 (48.8)	98 (40.8)	37 (45.1)	82 (40.8)
***p* value**	0.391 ^a^		0.553 ^a^		0.124 ^a^	
**Rh group (%)**						
**Pos**	93 (91.2)	164 (90.6)	39 (90.7)	218 (90.8)	69 (84.1)	188 (93.5)
**Neg**	9 (8.8)	17 (9.4)	4 (9.3)	22 (9.2)	13 (15.8)	13 (6.5)
***p* value**	0.874 ^a^		0.977 ^a^		0.013 ^a^	
**Laboratory parameters**						
**(Mean ± SD)** **WBC (cells/mm^3^)**	4360.1 ± 2698.1	5318.4 ± 3341.1	5053.7 ± 3657.8	4958.6 ± 3062.9	4939.88 ± 3062.3	4986.57 ± 3197.4
***p* value**	0.014 ^b^		0.856 ^b^		0.910 ^b^	
**PCV (%)**	42.5 ± 6.7	39.8 ± 6.3	41.2 ± 8	40.7 ± 6.3	39.8 ± 6	41.24 ± 6.7
***p* value**	**0.001 ^b^**		0.641 ^b^		0.097 ^b^	
**HB (g/dL)**	14.4 ± 2.5	13.2 ± 2.2	13.9 ± 2.7	13.5 ± 2.3	13.4 + 2.4	13.7 ± 2.3
***p* value**	**0.000 ^b^**		0.391 ^b^		0.358 ^b^	
**AST (U/L)**	147.4 ± 288.2	100.4 ±101.6	125.3 ± 147.7	115.97 ± 199	97.66 ± 112.6	125.4 ± 215.8
***p* value**	0.048 ^b^		0.770 ^b^		0.270 ^b^	
**ALT (U/L)**	73.1 ± 106.8	68.9 ± 70.4	63.6 ± 64.9	71.7 ± 88.3	57.11 ± 57.2	75.92 ± 93.8
***p* value**	0.693 ^b^		0.567 ^b^		0.092 ^b^	
**AST^2^/ALT**	338.5 ± 815.7	169.3 ± 189.8	274.5 ± 370.4	222.4 ± 540	188.8 ± 269.3	247.3 ± 589.4
***p* value**	**0.008 ^b^**		0.544 ^b^		0.389 ^b^	
**aPTT (sec)**	37.2 ± 8.5	32.7 ± 4.2	36.4 ± 6	34 ± 6.5	34 ± 6.3	34.5 ± 6.6
***p* value**	**0.000 ^b^**		0.026 ^b^		0.572 ^b^	
**PT (sec)**	14.8 ± 2	15 ± 1.8	15.2 ± 2.2	14.9 ± 1.8	15.5 ± 2.3	14.7 ± 1.6
***p* value**	0.410 ^b^		0.437 ^b^		**0.001 ^b^**	
**Platelet count (cells/mm^3^)**						
**(Mean ± SD)**	26,473.5 ± 29,109	126,093.92 ± 86,996.4	26,179.07 ± 27,351.1	101,656.7 ± 88,126.7	72,093.9 ± 77,515.6	97,570.15 ± 88,623
<10,000	20 (19.6)	1 (0.5)	7 (16.2)	14 (5.8)	11 (13.4)	10 (5)
10,001–20,000	46 (45.1)	9 (4.9)	21 (48.9)	34 (14.2)	20 (24.4)	35 (17.4)
20,001–50,000	24 (23.5)	30 (16.6)	10 (23.2)	44 (18.3)	14 (17)	40 (19.9)
50,001–100,000	8 (7.8)	44 (24.3)	4 (9.3)	48 (20)	14 (17)	38 (18.9)
>100,000	4 (3.9)	97 (53.6)	1 (2.3)	100 (41.7)	23 (28)	78 (38.8)
***p* value**	**0.000 ^b^**		**0.000 ^b^**		0.024 ^b^	
**Serological test (positive) (%)**						
**NS1**	93 (91.2))	128 (70.7)	39 (90.7)	182 (73.8)	69 (84.1)	152 (75.6)
***p* value**	**0.000 ^a^**		0.030 ^a^		0.116 ^a^	
**IgM**	36 (35.3)	97 (53.6)	13 (30.2)	120 (50)	31 (37.8)	102 (50.7)
***p* value**	**0.003 ^a^**		0.017 ^a^		0.048 ^a^	
**IgG**	13 (12.7)	3 (1.6)	5 (11.6)	11 (4.5)	4 (4.9)	12 (6)
***p* value**	**0.000 ^a^**		0.065 ^a^		0.718 ^a^	
**Length of hospital stay days)**	(6.5 ± 1.5)	(5.2 ± 2.2)	(6.6 ± 1.7)	(5.5 ± 2.1)		
**<6 days**	58 (56.9)	143 (79)	25 (58.1)	176 (73.3)		
**>6 days**	44 (43.1))	38 (21)	18 (41.9)	64 (26.7)	**_**	
***p* value**	**0.000 ^a^**		0.043 ^a^			

Statically significant, *p* < 0.01. ^a^ Chi-square test, ^b^ Levene’s independent *t* test conducted on different variables.

**Table 2:** Odds ratio of platelet transfusion vs. no platelet transfusion from laboratory parameters.

Parameter	n (%)	Category	Odds Ratio and *p* Value
NS 1	221(78)	Pos	4.279 (**<0.001** ^c^)
	62 (21.9)	Neg	
ASCITES	64 (22.6)	Yes	5.059 (**<0.001** ^c^)
	219 (77.4)	No	
GALL BLADDER WALL THICKENING/EDEMA	48 (17)	Yes	4.212 (**<0.001** ^c^)
	235 (83)	No	
PLEURAL EFFUSION	45 (15.9)	Yes	2.917 (**0.001** ^c^)
	238 (84)	No	
WBC **(cells/mm^3^)**	194 (68.5)	<5318	2.137 (**0.008** ^c^)
	89 (31.4)	>5318	
HB **(g/dL)**	111 (39.2)	>14.3	2.151 (**0.003** ^c^)
	172 (60.7)	<14.3	
AST2/ALT	127 (45)	>169.3	2.431 (**<0.001** ^c^)
	156 (55.1)	<169.3	
aPTT **(s)**	71 (25)	>37.2	5.815 (**<0.001** ^c^)
	211 (74.5)	<37.2	
Platelet count **(cells/mm^3^)**	96 (33.9)	<26,473.5	26.261 (**<0.001** ^c^)
	187(66)	>26,473.5	

Statically significant, *p* < 0.01. ^c^ Fisher’s exact test.

**Table 3:** Odds ratio of bleeding vs. no bleeding for various laboratory parameters.

Parameter	n (%)	Category	Odds Ratio and *p*-Value
aPTT **(secs)**	83 (29.3)	>36.4	3.074 (**0.002** ^c^)
	200 (70.6)	<36.4	
Platelet count **(cells/mm^3^)**	94 (33.2)	<26,179.07	8.352 (**<0.001** ^c^)
	189 (66.8)	>26,179.07	

Statically significant, *p* < 0.01. ^c^ Fisher’s exact test.

**Table 4:** Odds ratio of predictors of length of hospital stay.

Parameter	n (%)	Category	Odds Ratio and *p*-Value
PT **(secs)**	112 (39.6)	>15.5	2.432 (**0.001** ^c^)
	171 (60.4)	<15.5	

Statically significant, *p* < 0.01. ^c^ Fisher’s exact test was conducted for each category and for platelet transfusion, bleeding, and length of hospital stay.

## Data Availability

The data presented in this study are available upon request from the corresponding author.
